# Global analysis of transcription factors using both the reference genome and the multi-transcriptome of *Akebia trifoliata* reveals a sophisticated functional strategy

**DOI:** 10.3389/fpls.2025.1529326

**Published:** 2025-08-28

**Authors:** Hao Yang, Jie Li, Shengfu Zhong, Huai Yang, Rui Han, Xiaoxiao Yi, Xueying Li, Peigao Luo

**Affiliations:** ^1^ Key Laboratory of Plant Genetics and Breeding at Sichuan Agricultural University of Sichuan Province, College of Agronomy, Sichuan Agricultural University, Chengdu, Sichuan, China; ^2^ Rice Research Institute, Sichuan Agricultural University, Chengdu, Sichuan, China; ^3^ Industrial Crop Research Institute, Sichuan Academy of Agricultural Sciences, Chengdu, Sichuan, China

**Keywords:** transcription factors, *Akebia trifoliata*, TF cooperation, regulatory network, expression pattern

## Abstract

**Introduction:**

All the transcription factors (TFs) encoded by a genome collectively constitute a functional entity that closely regulates various life activities through mutual cooperation. *Akebia trifoliata*, which has great industrial and medicinal value, has significant potential as a model plant for use in perennial horticultural studies, and its TF repertoire must be comprehensively resolved.

**Methods:**

We identify all TFs from the *A. trifoliata* reference genome via DBD homology matching, subsequently characterizing their chromosomal distribution, gene structure, protein properties, and binding motifs, while further employing all available RNA-seq data to assess TF family conservation and infer potential interactions through co-expression analysis.

**Results:**

The TF repertoire of *A. trifoliata* consists of 1602 transcription factors from 56 families, revealing uneven chromosomal distributions and variations in gene structure, GC content, molecular weight, and evolutionary features such as duplication and selection strength. Functional annotation indicated that these TFs play diverse regulatory roles in various biological pathways, despite members within the same family often having similar functions. The expression profile data further supported the pleiotropic nature of many TFs, and their tissue-specific expression modules and functional enrichment were characterized. Notably, cooperative interactions were frequently observed within and across TF families, and some of these interactions were highly credible, as identified by co-expression analysis. Additionally, among all of them, variant sites were detected in 1473 TFs, whereas variant sites were not detected in 129 TFs.

**Discussion:**

This comprehensive analysis offers valuable insights into the regulatory mechanisms of *A. trifoliata*, enhancing our understanding of TF interactions and their roles in the development and adaptability of this organism.

## 1 Introduction

All the transcription factors (TFs) encoded by a genome collectively compose the key regulatory toolkit of a given organism; they are usually proteins in chemical essence while functionally controlling gene expression by directly recognizing and binding to DNA in a sequence-specific manner (De Mendoza et al., 2013). Early reports about specific proteins regulating gene expression date back to the classic lac operon model of 1961 (Jacob and Monod, 1961). In the 1980s, TF families such as the *C2H2-zinc finger* (*ZF*), *homeodomain basic helix-loop-helix* (*bHLH*) and *basic leucine zipper* (*bZIP*) families were generally reported in *Xenopus laevis* (Rosenberg et al., 1986), *Drosophila* (Villares and Cabrera, 1987) and rats (Landschulz et al., 1988), respectively, and in the 1990s, several new TF gene families, such as *AGAMOUS* (*AG*) and *SQUAMOSA PROMOTER BINDING PROTEIN-LIKE* (*SBP)*, were also systemically identified in the model plant *Arabidopsis thaliana* (Yanofsky et al., 1990; Cardon et al., 1997). A Science Direct search (https://www.sciencedirect.com/) on March 12, 2024 using the terms “plant” and “transcription factor” returned 97,268 research articles and 35,558 reviews, which suggested that TFs have attracted the widespread attention of many plant biologists.

Functionally, TFs exhibit significant pleiotropism in regulating various life activities. For example, the *MYB* family can simultaneously regulate many functional genes in the phenylpropanoid, anthocyanin, and flavonoid biosynthesis pathways (Pratyusha and Sarada, 2022). In eukaryotes, the pleiotropism of TFs largely depends on their cooperation, a sophisticated mechanism through which multiple TFs interact to increase both their specificity and diversity of binding to DNA simultaneously (Mahendrawada et al., 2025). Given the relatively limited number of TFs within a genome, they must generate numerous functional combinations to ensure the spatial and temporal specificity of the vast array of downstream functional gene expression. Therefore, identifying these TF combinations is crucial for elucidating their regulatory mechanisms, physiological functions, and even regulatory hierarchies.

The most common form of this interaction is the formation of TF dimers. Dimeric TF pairs typically recognize half of a core short palindrome (at least 6 bp) in the promoter region (Morgunova and Taipale, 2017) to increase their affinity for DNA (Todeschini et al., 2014). In terms of origin, these TFs can be either homologous or heterologous (Bemer et al., 2017), such as those in the *ERF* (Hou et al., 2020), *MADS* (Lai et al., 2019) and *bZIP* (Rodríguez-Martínez et al., 2017) families. With respect to their binding positions, they may be adjacent to or separated by a few bp on the same side of the DNA, forming intrastrand palindromes, or symmetrically distributed on opposite sides of the DNA, forming interstrand palindromes (Jolma et al., 2015). Therefore, short palindromes in TF binding motifs (TFBMs) can be used for the comprehensive identification of dimeric TF cooperation.

TFs are classified into different families based on the structural characteristics of their DNA-binding domains (DBDs). Almost all existing examples of DBDs are believed to have originated from the replication and evolution of a small group of common ancestors representing the major DBD folds (Lambert et al., 2018; Liang and Schnable, 2017). Compared with animals, plants have undergone more frequent genome duplication events (Shiu et al., 2005), leading to the evolution of many plant-specific TF families (Yamasaki et al., 2008). Studies have shown that throughout the evolution of the streptophyte lineage, the emergence of specialized plant organs such as stomata, vascular tissues, roots, reproductive cones, or flowers has depended on repeated family expansions involving specific TFs (Lai et al., 2020). As species have evolved, these functions have been consistently retained. For example, plant-specific TF families have been shown to broadly regulate the development of organs such as seeds, leaves, meristems, and flowers, and they also participate in secondary metabolism and hormone responses to adapt to environmental changes (Lehti-Shiu et al., 2017). These findings indicate that plant-specific TFs are indispensable for normal plant growth and development and that the evolutionary traces of these families offer significant insights for further research.

*Akebia trifoliata*, a widely distributed woody vine across East Asia, Europe, and North America (Huang et al., 2022), holds significant value for fruit, medicinal herbs, cosmetic ingredients, and industrial material development. Evolutionarily, *A. trifoliata* is classified as a basal eudicot, representing a transitional state between basal angiosperms and core eudicots; morphologically, it is in a transitional state between herbaceous and woody plants. These characteristics are advantageous for studying the functional differentiation of TF families. Additionally, *A. trifoliata* has a small genome (675 Mb), a short juvenile phase, and a high seed yield, making it an ideal material for studying TF repertoires in perennial horticultural plants (Zhong et al., 2022a). Although some studies have focused on individual TF families in *A. trifoliata*, including *Dof* (Zhang et al., 2023), *NAC* (Liu et al., 2023) and *WRKY* (Wen et al., 2022; Zhu et al., 2022), these studies have overlooked the interactions between different families, and a comprehensive understanding of the formation of *A. trifoliata* TF repertoires is still lacking. However, the available reference genomes and numerous RNA sequencing datasets offer valuable opportunities to study TF function and variation comprehensively.

In this study, we used the Plant Transcription Factor Database (https://planttfdb.gao-lab.org/) based on DBD homology matching to identify all possible TFs from the reference genome of *A. trifoliata* and analyzed their chromosomal distribution, gene structure, protein characteristics, and binding motifs. Additionally, we used all the available RNA-seq data for *A. trifoliata* to analyze the conservation of various TF families and to identify potential TF interactions on the basis of co-expression patterns.

## 2 Materials and methods

### 2.1 Identification of TFs from the *A. trifoliata* genome

The *A. trifoliata* reference genome with BioProject PRJNA671772 in NCBI was employed because of its detailed annotation and assembly quality (Zhong et al., 2022). All the protein sequences of the TFs were annotated in the Plant Transcription Factor Database v5.0 using the prediction tool on Nov. 13^th^, 2023. The database consisted of 165 plant genomes (100 eudicots, 38 monocots, other 7 older angiosperms and 20 lower plants), in which all the TFs were classified into 58 families (Jin et al., 2017).

### 2.2 Characterization of the basic information on all the TFs

The chromosome distribution, gene structure (including the gene length, exon number, and GC content) and protein features (including the amino acid number, molecular weight, and theoretical pI) were analyzed using the bioinformatics software TBtools v2.302 (Chen et al., 2020). The inference of gene duplication events from these TFs, including five duplication types of “whole-genome duplication (WGD) or segmental”, “dispersed”, “tandem” “proximal” and “singleton”, was also analyzed using TBtools. TF clustering was performed with a 250-kb nonoverlapping sliding window across each chromosome to identify genomic regions containing 2 or more TF-encoding genes as primary clusters. Adjacent primary clusters were merged into final merged clusters if they shared at least one TF family member, ensuring a contiguous distribution of TF clusters. The collinear TF pairs and their Ka/Ks values were detected using the TBtools plugin in One Step MCScanX. When calculating the average Ka/Ks values for each family, families with fewer than two members were excluded. The subcellular localization of TFs was predicted on the website http://www.softberry.com using the ProtComp v9.0 tool. The N-linked glycosylation sites were predicted on the website https://services.healthtech.dtu.dk using the NetNGlyc v1.0 tool. The secondary structures of the TF proteins were identified in the Jpred 4 database (Drozdetskiy et al., 2015) (https://www.compbio.dundee.ac.uk/jpred).

### 2.3 KEGG and GO functional annotation

All the TFs were annotated for KEGG and GO function terms using their protein sequences with the EGGNOG-MAPPER database (http://eggnog-mapper.embl.de). The second- and fifth-level GO annotation terms used in the subsequent analysis were queried in OBO-Edit2 software v2.3.1 using the go-basic.obo file.

### 2.4 Identification of TFBMs and their distribution in promoter regions

To understand the potential DNA-binding sequence of these TFs, we inferred the TFBMs of *A. trifoliata* TFs by homology comparison with *A. thaliana* TFs. First, we obtained experimentally validated TFBMs (in MEME format) of all the *A. thaliana* TFs derived from 33 independent ChIP-seq datasets of *A. thaliana* in the PlantTFDB (https://planttfdb.gao-lab.org/download.php). We subsequently performed a homology comparison between *A. trifoliata* TFs and *A. thaliana* TFs using only the CDS region, matching each *A. trifoliata* TF with the *A. thaliana* TF with the lowest e value (threshold = 0.001), and we assigned the TFBM of the matched *A. thaliana* TF to the corresponding *A. trifoliata* TF. We further used the Find Individual Motif Occurrences (FIMO) analysis function in TBtools software to scan the promoter regions (500 bp upstream) of both the 1,602 TFs and 22,536 other genes across the genome (regarded as structural genes (SG)) with the obtained TFBM MEME files using the same e-value threshold.

### 2.5 Identification of short palindromes between TFBMs

To detect the possible interactions between any two TFs and the predicted TFBMs, we used python scripts to search for TFBM fragments in both TFs that could form a complete palindrome, in which the palindrome length was at least 6 bp (Datta and Rister, 2022). Once fragments in the TFBM of a specific pair of TFs could constitute palindromes, they were considered to have interaction potential. This search also included cases for which the same TF interacts with itself.

### 2.6 Acquisition and assembly of RNA-seq samples from multiple tissues

To characterize the expression patterns of these TFs in different tissues, a total of 90 RNA-seq samples from six BioProjects and four tissues in NCBI were used in our analysis. These samples consisted of 9 samples of peels in PRJNA524995 (Niu et al., 2020), 15 samples of seeds in PRJNA685604 (Huang et al., 2021), 12 samples of seeds in PRJNA792843 (Huang et al., 2021; Zhong et al., 2022b), 9 samples of seeds in PRJNA884501 (Liu et al., 2023), 9 samples of leaves in PRJNA1098036, and 36 samples of various tissues in PRJNA671772 (Zhong et al., 2022c).

We obtained the raw data for each program in SRA file format, first converted the SRA files to fastq files using fastq-dump, then removed any adapters and low-quality data using fastp (filter parameter: -m 20 -l 36 -n 0 -q 20 -3 20 -5 20), and lastly mapped the fastq files of different transcripts to the *A. trifoliata* reference genome using hisat2. The alignment results were saved as sequence alignment/map (SAM) files, which were consequently converted to binary alignment/map (BAM) files using SAMtools. The reads within each transcript were reordered for subsequent analysis.

### 2.7 Variant site identification and annotation of multiple transcripts

The BAM files of transcripts from each project were further converted to Variant Call Format (VCF) files, merged, and then applied to variation site extraction using BCFtools. The variant sites of all the TFs were separately extracted from each program individually, except PRJNA671772, which required three extractions due to the presence of three tissues. The variation rates of the CDSs, introns and UTRs from each project were calculated as follows: (total variation sites of region)/(region length×sample counts of each project). The variation rates of the three regions of each family were calculated as follows: (total variation sites of region)/(region length×90×family member with corresponding region). All the variant sites were annotated on the reference genome using ANNOVAR (Wang et al., 2010).

### 2.8 Expression profile analysis of all the TFs

The merged transcripts were used to determine the TPM gene expression level using stringtie. In addition, a weighted gene co-expression network analysis (WGCNA) was performed using the R package available at https://github.com/ShawnWx2019/WGCNA-shinyApp. TFs with transcripts per million (TPM) levels of less than 1 in 90% of the samples were filtered out before the expression data were input (Yang et al., 2024). Two calculation parameters, the R^2^ cutoff and module cutoff tree height, were set to 9 and 0.25, respectively. For expressed TFs, the coefficient of variation (CV) of expression was calculated as the mean of its expression in 90 samples divided by the standard deviation.

## 3 Results

### 3.1 Component and chromosomal distributions of all the TFs in the reference genome

**Figure 1:**
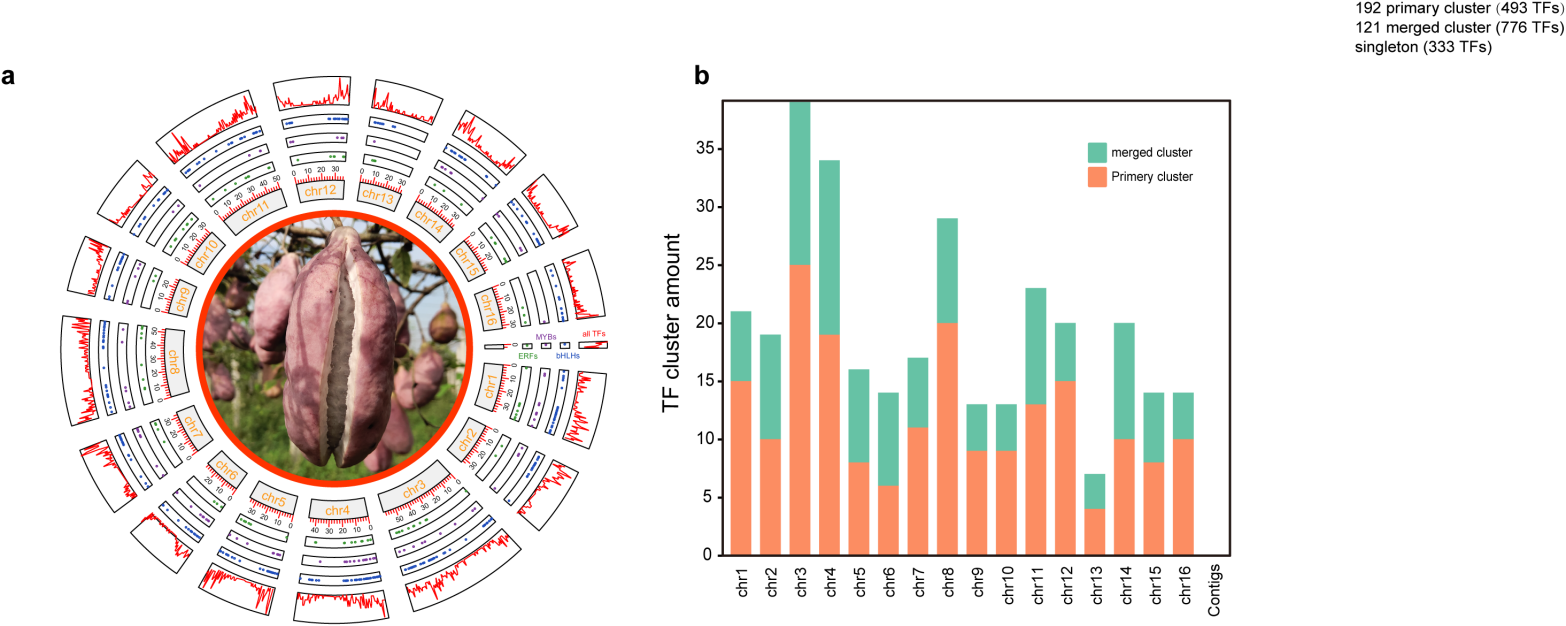
Distributions of TF and TF clusters among chromosomes. (a) Distributions of *ERFs*, *MYBs*, *bHLHs* and all the TFs among 16 chromosomes or contigs from inside to outside. (b) The distribution of TF clusters in each chromosome. TFs on unassembled contigs were not counted.

A total of 1602 TFs were systemically identified in the *A. trifoliata* reference genome (Zhong et al., 2022b), and their detailed information is listed in **Supplementary Table S1**. These proteins consisted of almost all 58 types except *nuclear transcription factor X-box binding 1* (*NF-X1*) and *SQUAMOSA promoter-binding protein* (*SBP*) (**Table 1**). The members of each gene family exhibited large variation, and the top three gene families were *basic helix-loop-helix domain* (*bHLH*), *MYB domain* (*MYB*) and *ethylene response factor* (*ERF*), with 168, 126 and 109 members, respectively, whereas the number of gene families with fewer than 10 members was 20 (35.7%); there was only one member each in *LEAFY protein* (*LFY*) and *signal transducer and activator of transcription* (*STAT*) (**Table 1**). Among the 1602 TFs, 1,560 (97.4%) could be precisely mapped to 16 chromosomes, and only 42 (2.6%) were assigned to the unassembled scaffold contigs (**Supplementary Table S1**); notably, they were distributed primarily in the proximal regions of each chromosome (**Figure 1A**). The numbers of TFs on chromosomes 3 and 13 were the highest (166, 10.4%) and the lowest (54, 3.4%), respectively (**Supplementary Table S2**). In addition, there was a significant correlation (R = 0.978, p < 0.001) between the TF number and the chromosomal length (**Supplementary Table S2**).

**Table 1:** Characteristics of 56 TF families in *Akebia trifoliata*.

TF Family	Gene feature			Protein feature			Secondary structure			Sl	
	Length(kbp)	Exon number	GC%	AA	MW (kDa)	pI	E	H	O1	N	O2
AP2 (26)	4.7	8.5	36.3	457.8	51.	7.3	7.5	4.6	12.5	25	1
ARF (27)	7.8	12.0	38.2	795.0	88.4	6.3	19.7	9.5	27.8	27	0
ARR-B (18)	6.5	5.8	35.4	657.9	73.2	5.8	6.7	13.3	20.4	8	10
B3 (37)	7.0	5.2	36.3	433.9	49.1	7.9	13.3	6.4	20.0	21	16
BBR-BPC (8)	2.7	2.0	39.8	307.0	34.1	9.5	4.3	3.9	9.0	8	0
BES1 (8)	5.9	5.3	37.8	437.8	48.6	7.6	7.0	6.9	14.6	5	3
bHLH (168)	3.8	4.5	36.6	335.9	37.5	6.8	3.2	4.4	8.3	115	53
bZIP (65)	7.7	5.3	36.5	341.1	37.8	7.1	1.4	4.0	6.3	39	26
C2H2 (89)	3.4	2.9	39.2	408.0	45.5	7.7	12.4	4.2	17.0	44	45
C3H (51)	9.4	5.4	37.6	509.4	56.5	7.2	4.5	6.5	11.4	23	28
CAMTA (7)	14.9	12.4	36.2	956.4	107.9	6.1	12.6	21.3	34.7	7	0
CO-like (10)	3.8	3.4	38.3	400.7	44.8	5.7	5.4	4.3	10.2	10	0
CPP (8)	8.9	9.9	36.3	736.0	81.1	6.6	4.8	6.3	11.5	6	2
DBB (7)	6.5	3.7	37.6	256.7	28.3	5.9	5.1	1.4	7.6	7	0
Dof (41)	2.0	1.7	39.3	300.8	33.2	8.1	1.2	0.6	2.8	30	11
E2F/DP (11)	6.9	11.2	36.7	373.2	41.9	7.5	8.5	8.2	16.0	11	0
EIL (2)	3.3	2.5	40.0	600.0	67.8	5.4	7.5	11.0	19.5	2	0
ERF (109)	2.1	1.5	41.2	254.4	28.4	6.6	3.5	2.0	6.3	87	22
FAR1 (34)	8.5	4.5	36.5	584.9	67.1	7.1	12.2	17.1	28.8	8	26
G2-like (46)	5.0	5.5	35.8	343.3	38.5	6.7	1.3	4.8	7.2	19	27
GATA (36)	6.2	4.5	36.7	301.2	33.3	7.3	2.1	2.3	5.3	28	8
GeBP (8)	1.7	1.0	39.9	353.1	40.4	7.0	0.1	6.3	7.4	0	8
GRAS (52)	2.7	1.6	42.3	540.0	60.5	5.6	8.7	13.2	22.5	35	17
GRF (10)	3.7	3.9	37.7	410.5	45.5	8.0	1.3	2.9	5.2	9	1
HB-other (12)	11.3	9.8	36.9	714.9	80.5	7.5	6.1	14.4	21.3	11	1
HB-PHD (2)	12.0	10.5	36.5	999.0	111.4	7.6	30.5	9.0	39.5	2	0
HD-ZIP (44)	4.6	7.3	36.7	455.8	50.9	6.3	8.7	9.3	18.0	44	0
HRT-like (4)	13.0	3.3	36.3	540.5	60.8	9.3	17.0	8.0	25.8	0	4
HSF (24)	3.4	2.8	36.9	372.6	42.3	6.1	4.3	6.6	11.5	21	3
LBD (35)	2.1	2.2	39.0	217.5	24.1	7.1	0.9	5.4	7.2	25	10
LFY (1)	4.4	3.0	38.4	378.0	42.6	7.1	3.0	13.0	17.0	1	0
LSD (2)	10.8	5.5	33.9	145.0	15.5	8.3	9.0	0.0	10.0	2	0
MIKC_MADS (26)	17.2	7.9	34.0	237.4	27.3	8.2	2.8	5.2	8.5	26	0
M-type_MADS (20)	4.2	4.1	38.5	258.1	29.3	7.7	3.3	5.9	9.8	14	6
MYB (126)	3.0	3.3	36.7	371.8	41.9	7.0	0.8	7.8	9.5	95	31
MYB_related (59)	7.3	6.7	36.4	469.8	52.5	7.8	3.1	8.5	12.2	32	27
NAC (93)	3.6	4.0	36.0	359.3	40.9	6.5	8.3	2.1	11.2	62	31
NF-YA (7)	13.7	6.1	35.8	304.6	33.5	8.9	1.9	2.7	5.6	3	4
NF-YB (14)	5.6	3.4	37.8	186.5	20.5	5.8	0.5	5.4	6.9	13	1
NF-YC (13)	5.5	3.5	37.6	220.4	24.8	6.1	0.2	5.6	6.8	9	4
Nin-like (15)	6.8	4.7	36.5	547.5	61.2	6.0	9.5	11.6	21.3	11	4
NZZ/SPL (4)	1.1	3.5	40.7	238.8	26.6	9.4	3.5	2.3	6.8	0	4
RAV (2)	1.4	1.0	40.7	360.5	40.8	7.7	10.0	6.0	15.5	2	0
S1Fa-like (3)	4.1	2.3	36.6	290.7	31.7	9.0	6.0	6.0	13.0	2	1
SAP (25)	8.3	5.4	37.8	542.5	60.4	7.7	8.8	9.0	17.8	21	4
SRS (5)	2.2	2.0	40.5	301.2	32.8	8.2	6.8	0.8	8.4	5	0
STAT (1)	12.2	10.0	35.3	515.0	56.9	5.3	22.0	8.0	31.0	0	1
TALE (24)	9.5	5.0	36.1	514.9	57.4	6.0	2.5	9.0	12.5	24	0
TCP (26)	3.1	1.7	40.4	359.4	39.5	7.2	4.1	3.7	8.5	17	9
Trihelix (41)	4.8	2.7	38.9	394.1	44.8	7.5	2.0	8.0	10.8	11	30
VOZ (3)	6.1	4.0	36.4	443.3	49.3	5.6	6.3	8.0	15.3	0	3
Whirly (2)	10.8	7.5	36.7	274.0	30.6	9.6	10.5	3.0	14.0	0	2
WOX (10)	3.0	3.0	36.9	257.7	29.3	7.5	2.1	4.1	7.0	10	0
WRKY (65)	3.9	3.9	35.9	385.8	42.9	7.1	6.2	2.0	8.9	52	13
YABBY (6)	4.7	6.7	34.1	186.2	20.9	8.4	3.5	2.5	6.8	6	0
ZF-HD (10)	1.6	1.3	39.8	254.3	28.3	7.9	3.6	3.8	7.6	9	1

AA represents the average amino acid residue number; MW (kDa) represents the molecular weight in thousands of daltons; and pI represents the theoretical isoelectric point. E, H and O1 represent extended, helical and other secondary structures, respectively. The SI represents the subcellular localization prediction, and N and O2 represent the nucleus and other sites, respectively.

Further sliding window analysis revealed that 1,602 TFs exhibited 333 singlets, 192 primary clusters with 493 TFs from 48 gene families within the 250-kb length and 121 merged clusters with 776 TFs from 50 gene families in regions greater than 250-kb length, and the singlets, primary and merged clusters were widely distributed across all the chromosomes (**Supplementary Table S3**). Chromosomes 3 and 13 had the most (39—25 primary and 14 merged) clusters and the fewest (7—4 primary and 3 merged) clusters, respectively (**Figure 1B**; **Supplementary Table S3**), and the longest TF cluster, with a length of 1.58 Mb on chromosome 16, contained 23 TFs from 15 families, whereas the shortest TF cluster, with a length of only 4.7 kb on chromosome 16, contained only 2 TFs (*Trihelix6* and *B3-5*) (**Supplementary Table S3**). Except for the *STAT* (1 member) and *LSD* (2 members) families, at least one member of the remaining 54 gene families was distributed on chromosomes in clusters. Moreover, all 42 members of 10 families, including *BBR-BPC, CAMTA, EIL, HB-PHD, LFY, RAV, SRS, VOZ, whirly* and *ZF-HD*, were distributed in clusters (**Supplementary Table S3**). In addition, 23 clusters were composed of TFS from the same family, of which 4 clusters were composed of 3 members from the same family (*B3, ERF, FAR, HSF*), and 19 clusters were composed of 2 genes from the same family (*bHLH, GeBP, LBD, MYB_related, MYB, NAC* and *WRKY*). The members within any one of all 119 merged clusters were from at least two different gene families, but their component proportions clearly differed. For example, 7 of the 9 members within the cluster in the 8682315–8955998 (273 kb) region of chromosome 11 came from the *NAC* family, whereas all 9 members within the cluster in the 8825047–4340861 (901 kb) region of chromosome 4 came from nine different families (**Supplementary Table S3**).

### 3.2 Characteristics of all the TFs and their putative proteins

Both the total length and exon number of the TFs exhibited large variations from 305 bp (*GRAS33*) to 80,166 bp (*MIKC_MADS10*) and from 1 in 268 TFs to 25 in *MYB_related24*, respectively; there was also an obvious variation in the GC content, from 29.4% (*MIKC_MADS8*) to 52.9% (*C2H2-35*) among the 1,602 TFs (**Supplementary Table S1**). Almost half (790, 49.3%) of the TFs had a length of less than 3,000 bp (**Supplementary Table S1**). In addition, the coefficients of variation (CVs) in gene length, number of exons, and GC content for 87%, 93%, and 95% of TF families were respectively lower than the CVs of all TFs in the corresponding aspects (**Supplementary Table S1**). The mean gene length of *NZZ/SPLs* was the shortest (1,062.3 bp) among the various families, whereas that of *MIKC_MADSs* was the longest (17,163.8 bp) (**Table 1**). The exons of some members of five families, including *ARF*, *CAMTA*, *E2F/DP, HB-PHD* and *STAT*, with only one member, numbered up to ten or more, whereas all ten members of both *GeBP* and *RAV* had only one exon (**Supplementary Table S1**). In addition, there were significant relationships between the GC content and both gene length (R = -0.31, P<0.0001) and exon number (R= 0.54, P<0.0001) (**Supplementary Table S5**).

For the characteristics of the putative proteins, there were also large variations in the amino acid residue number, ranging from 73 (MYB_related 3) to 1,755 (HB-other 9); in molecular weight, ranging from 8,375.2 (MYB_related 3) to 196,052.8 (HB-other 9); and in the theoretical isoelectric point, ranging from 4.36 (M-type_MADS4) to 10.75 (NAC31), of all the TFs (**Supplementary Table S1**). We found that 46 and 44 TF families presented smaller CVs in amino acid number and isoelectric point, respectively, than did all the other TF families (**Supplementary Table S4**). In addition, the LSD and HB-PHD families presented the smallest (145.0) and largest (999.0) average amino acid residue numbers (**Table 1**), respectively, and the STAT and the HB-PHD families presented the smallest (5.27) and largest (9.62) average isoelectric points, respectively (**Table 1**). Most (55, 98.2%) TF families simultaneously contained all three secondary structures (extended, helical and others), but the LSD family had no helical structure (**Table 1**), in which 34 (61.8%) families had more helical structures than extended structures. Similarly, variations in the proportions of the “extended”, “helical” and “other” structures also occurred primarily among different TF families (**Supplementary Table S4**). In addition, given that the 56 TF families were artificially divided into three groups with fewer than 10 members, from 10–30 members and larger than 30 members, the third group, with the greatest number of members, had a significantly lower average number of all three structures than the other two groups did (**Supplementary Table S6**). Many (1,104, 68.9%) TFs were predicted to be located in the nucleus. Among them, all 194 members of 17 TF families, such as MIKC_MAD and WOX, were located in the nucleus, whereas all 22 members of 6 TF families (GeBp, HRT-like, NZZ/SPL, STAT, VOZ and whirly) were not located in the nucleus (**Table 1**, **Supplementary Table S1**).

### 3.3 Duplication events experienced by all the TFs

**Figure 2:**
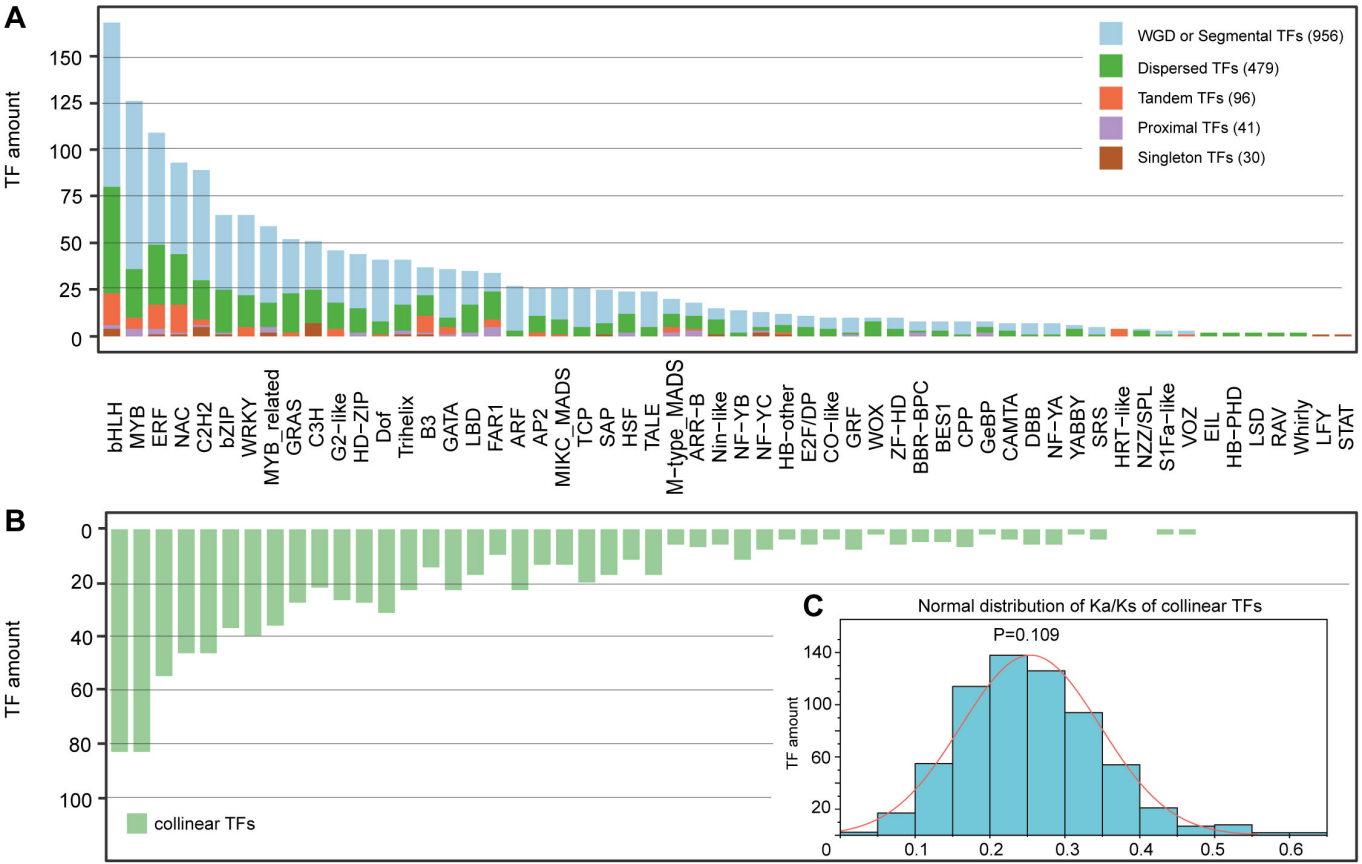
Distribution of duplication types and collinear TFs among 56 families. **(A)** Duplication types. **(B)** Collinear TFs. This figure shares the same horizontal coordinate with **(A, C)** The normal distribution of Ka/Ks of collinear TFs.

Among the 1602 identified TFs, 956 (59.7%), 479 (29.9%), 96 (6.0%) and 41 (2.5%) were produced by whole-genome duplication (WGD) or segmental, dispersed, tandem and proximal duplications, respectively; the remaining 30 (1.9%) TFs were singlets (**Figure 2A**; **Supplementary Table S7**). Except for two families (*LFY* and *STAT*) with only one member, five families (*EIL, HB-PHD, LSD, RAV* and *whirly*) with two members and one family (*HRT-like*) with four members, the remaining 48 (85.7%) families evolutionarily experienced two or more duplication types (**Supplementary Table S7**). Additionally, the percentages of whole-genome duplication (WGD) and segmental duplication showed considerable variation, ranging from 0% in seven families (including two families with only one member and five families with two members) to 89% in the ARF family with 27 members. The percentage of dispersed duplication ranged from 0% in four families (HRT-like, LFY, STAT, and VOZ) to 100% in all five families with two members. Tandem duplication percentages varied from 0% in 37 families to 100% in the HRT-like family with four members. Proximal duplication percentages ranged from 0% in 36 families to 25% in both the BBR-BPC family and GeBP family, each containing eight members (**Table 1**; **Supplementary Table S7**). Interestingly, among the 26 families with more than 20 members, 25 (96.2%) were produced primarily by WGD or segmental duplication, and the percentages of TFs produced by WGD or segmental duplication were greater than 50% in 23 (88.5%) families, whereas only *FAR1*, with 34 members, experienced dispersed duplication (**Supplementary Table S7**).

### 3.4 Determination of collinearity and the corresponding Ka/Ks value

We found that 919 (58.1%) of the 1602 genes had homologs within the same families and did not find collinearity among members from different families (**Figure 2B**). The 919 collinear TFs produced a total of 640 homogenous gene pairs with varying numbers in each family, ranging from only one pair in five families (*GeBP3* and *GeBP5*, *S1Fa-like2* and *S1Fa-like3*, *VOZ2* and *VOZ3*, *WOX1* and *WOX8*, and *ABBY4* and *YABBY6*) to 69 pairs formed by 88 members in the *bHLH* family (**Supplementary Table S7**). In addition, the proportion of TFs with homologous counterparts among total TFs also exhibited large variation among various families, ranging from 20.0% in the *WOX* family to 88.9% in the *ARF* family (**Supplementary Table S7**). Lastly, among the 919 TFs, those with one, two, three, four and five homologs numbered 605, 239, 72, and 2 (*Dof1* with *Dof4*, *Dof19*, *Dof24* and *Dof35*, and *MYB40* with *MYB26*, *MYB75*, *MYB81* and *MYB120*) and 1 (*ARF26* with *ARF3*, *ARF17*, *ARF18*, *ARF22* and *ARF23*), respectively (**Supplementary Table S8**).

The calculated Ka/Ks values among the 640 homogenous gene pairs revealed that almost all the Ka/Ks values were much lower than 1, and only the Ka/Ks value of the *GRAS1*–*GRAS29* pair was 2.344, which was greater than 1. The Ka/Ks ratio exhibited normal patterns between 0.04 and 0.60, with the majority of values falling within the 0.2–0.25 range (**Figure 2C**; **Supplementary Table S7**). The average Ka/Ks of each family also varied from 0.124–0.366 (**Supplementary Table S7**), indicating strong purifying selection acting on most TF families.

Collectively, the widespread occurrence of Ka/Ks < 1 among collinear TF pairs demonstrates that most duplicated TF genes in *A. trifoliata* have undergone purifying selection, maintaining functional conservation. These findings align with the predominance of WGD in TF expansion, as WGD-derived genes often experience subfunctionalization under purifying selection (Liang and Schnable, 2017).

### 3.5 Functional annotation of TFs

**Figure 3:**
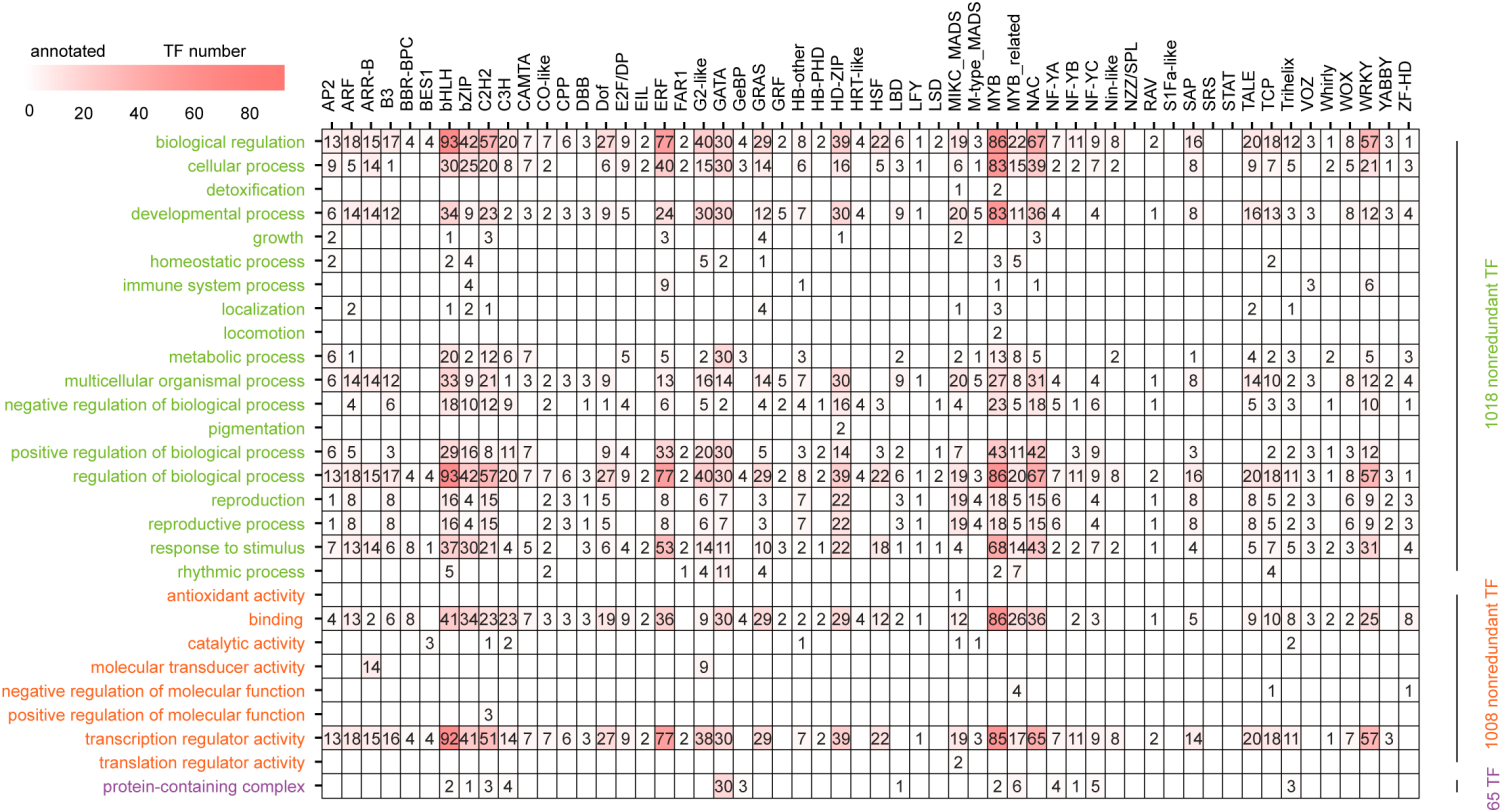
Distribution of secondary GO term annotations among 56 families. Biological processes, molecular functions and cellular components are marked in green, orange and purple, respectively.

In the Gene Ontology database, which consists of 3 primary terms with 61 secondary GO terms, 1,040 (64.9%) of all 1,602 TFs from all 56 families except for *NZZ/SPL, S1Fa-like, SRS* and *STAT* with four, three, five and one member(s), respectively, were annotated 6,858 times in the three primary terms and 28 secondary GO terms (**Supplementary Table S9**). Among them, 5,214 (76.0%), 1,579 (23.0%) and 65 (1.0%) annotations were produced by 1,018, 1,008 and 65 of the 52 annotated families, respectively, except the LSD family, with two members and 13 families in biological process, molecular function and cellular component primary GO terms (**Figure 3**; **Supplementary Table S9**). We further found that these annotations were distributed into 19 (82.6%), 8 (22.9%) and 1 (protein-containing complex, GO:0032991) (33.3%) of the corresponding 23, 35 and 1 (GO:0110165, GO:0032991 and GO:0044423) secondary GO terms, respectively. Among the 19 annotated secondary GO terms, 985 (96.8%) and 982 (96.5%) of the 1,018 TFs could be annotated as GO:0065007 (biological regulation) and GO:0050789 (regulation of biological process), respectively. Similarly, among the 8 secondary GO terms related to molecular function, GO:0140110 (transcription regulator activity) had the greatest number of TFs (933, 92.6%) (**Supplementary Table S9**).

Among the 52 annotated families, the members of 13 families could be annotated in all three primary GO terms. The number of secondary GO terms in various families varied from five in four families (BBR-BPC, BES1, HRT-like and LSD) to 20 in the MYB family. In addition, some annotated secondary GO terms were TF family-specific. For example, pigmentation (GO:0043473) and locomotion (GO:0040011) were annotated only by the HD-ZIP (*HD-ZIP9* and *HD-ZIP33*) and MYB (*MYB30* and *MYB45*) families, respectively (**Supplementary Table S9**).

In the KEGG database, only 220 (13.7%) TFs from 31 (55.4%) families were successfully annotated in 88 metabolic pathways, of which 31 (35.2%) annotated metabolic pathways contained only one TF, whereas “plant hormone signal transduction” (Ko 04075) contained the most (82, 37.3%) TFs from nine families (*ARF, ARR-B, BES1, bHLH, bZIP, EIL, ERF, G2-like* and *GRAS*) (**Supplementary Table S10**). Moreover, among the 88 pathways, 56 (63.6%) were family specific, and the number of TF families associated with the remaining 32 pathways ranged from 2–10 in the Ko01100 metabolic pathways. Among the 31 annotated families, ten families (*ARF, ARR-B, C2H2, CO-like, CPP, Dof, G2-like, GRAS, TCP*, and *WOX*) were annotated in only one pathway, whereas the E2F/DP family was assigned to 22 metabolic pathways (**Supplementary Table S10**).

Totally, the functional annotation reveals that *A. trifoliata* TFs primarily orchestrate transcriptional regulation and plant hormone signaling, with distinct families specializing in processes like development (HD-ZIP), stress response (ERF), and pigment synthesis. The enrichment of TFs in hormone pathways and family-specific GO and KEGG terms demonstrates their hierarchical roles in regulatory networks, balancing conserved transcriptional functions with lineage-specific adaptations.

### 3.6 Distribution of TFBMs in the promoter regions

**Figure 4:**
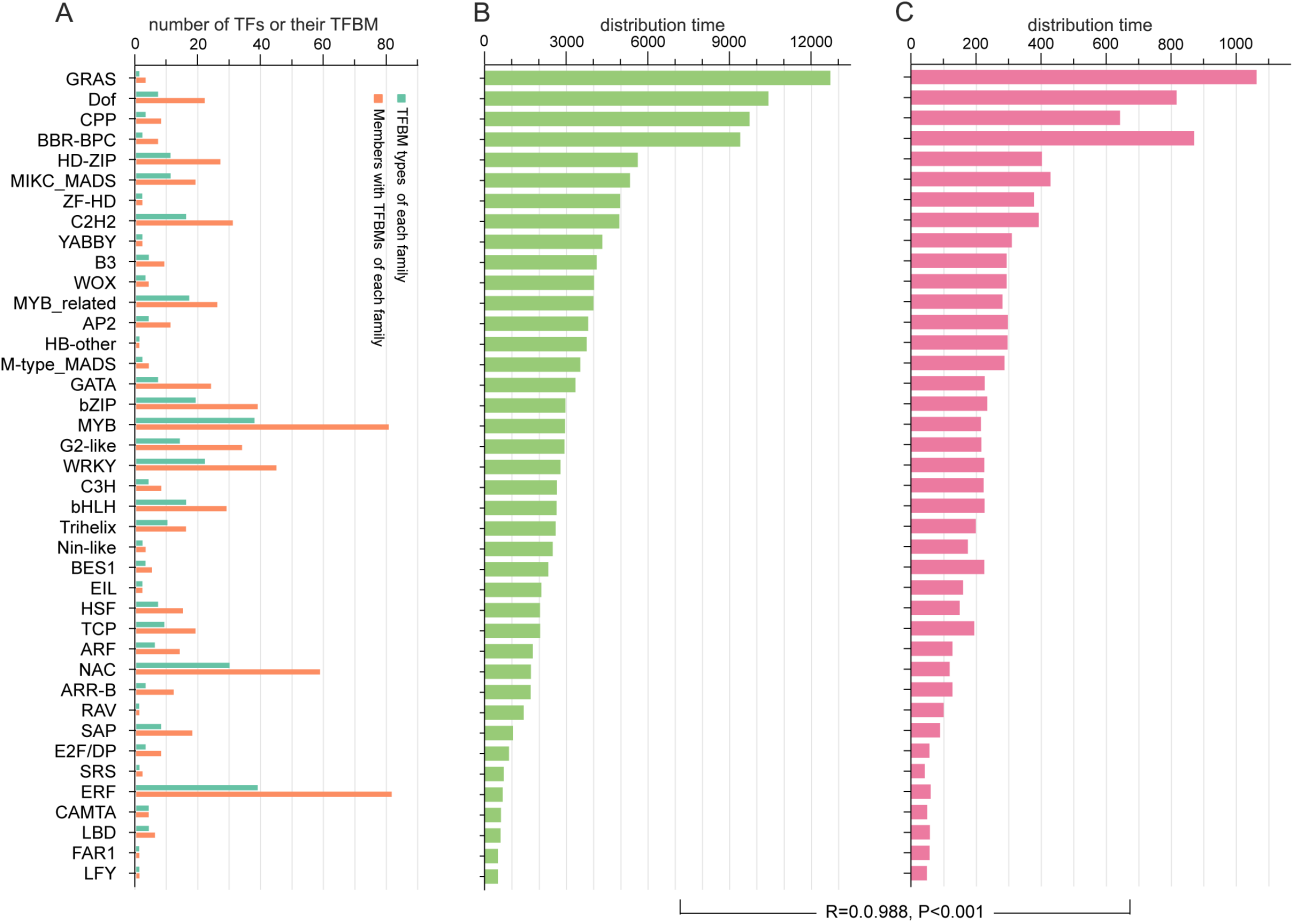
Distribution of TFBMs in the promoter regions of SGs and TFs in the *A. trifoliata* genome. **(A)** Distributions of TFs and their TFBMs in each family. **(B)** Average distribution times of each TF family in the promoter region of *A. trifoliata* structural genes. **(C)** Average distribution times of each TF family in the promoter region of *A. trifoliata* TFs.

A total of 331 (53.5%) of the 619 TF binding motifs (TFBMs) in *A. thaliana* TFs were assigned to 704 (43.9%) TFs from 40 families of *A. trifoliata*, among which 322 TFBMs were only shared by members within the same TF family and 9 TFBMs were shared by TFs across two families in *A. trifoliata* (**Supplementary Table S11**). For example, MP00054 was simultaneously shared by 9 ARR-B members and 2 G2-like members (**Supplementary Table S11**). Among the 40 families, the number of members with both TFBM and TFBM types in each family varied extensively, ranging from 1 (*FAR1, HB-other, LFY*, and *RAV)* to 82 (*ERF*) (**Figure 4A**) and from 1 (*FAR1, GRAS, HB-other, LFY, RAV*, and *SRS*) to 39 (*ERF*) (**Figure 4A**; **Supplementary Table S11**).

We further found that the 331 TFBMs were widely distributed 167,330 and 2,176,612 times in the −500 bp promoter regions of all 22,536 SGs and all 1,602 TFs (**Supplementary Table S11**). In addition, the average distribution time, which ranged from 482.0 (*LFY*) to 12,702.0 (*GRAS*) (**Figure 4B**) in all SGs, was markedly longer than that in all 1,602 TFs, which ranged from 42.0 (*SRS*) to 1,062 (*GRAS*) (**Figure 4C**), whereas their change tendency exhibited a significant relationship (R = 0.988, P<0.001) (**Figures 4B, C**), in which the average distribution times of the TFBMs in four families (*GRAS, Dof, CPP* and *BBR-BPC*) in both SGs and all 1,602 TFs were much greater than those in the other 36 families (**Supplementary Table S11**; **Figures 4B, C**). Interestingly, the TFBMs of 145 (20.6%) TFs from 28 (70%) families were also found in their own promoter regions (**Supplementary Table S12**). For example, the TFBM with the sequence 5’-AAAGATCAAAATAAGAGAAG-3’ of *AP2–4* was also found in the range from 332 bp to 313 bp on its own promoter (**Supplementary Table S12**). In addition, the proportion of self-regulating TFs was obviously greater in the *BBR-BPC* (37.5%) and *MIKC_MADS* (42.3%) families than in the other families (**Supplementary Table S12**).

These results indicate that A. trifoliata TFs employ both intra-family and cross-family TFBMs for regulatory network construction, with self-regulation potentially mediating transcriptional feedback loops to fine-tune gene expression.

### 3.7 Variation sites of TFs among various transcriptomic datasets

**Figure 5:**
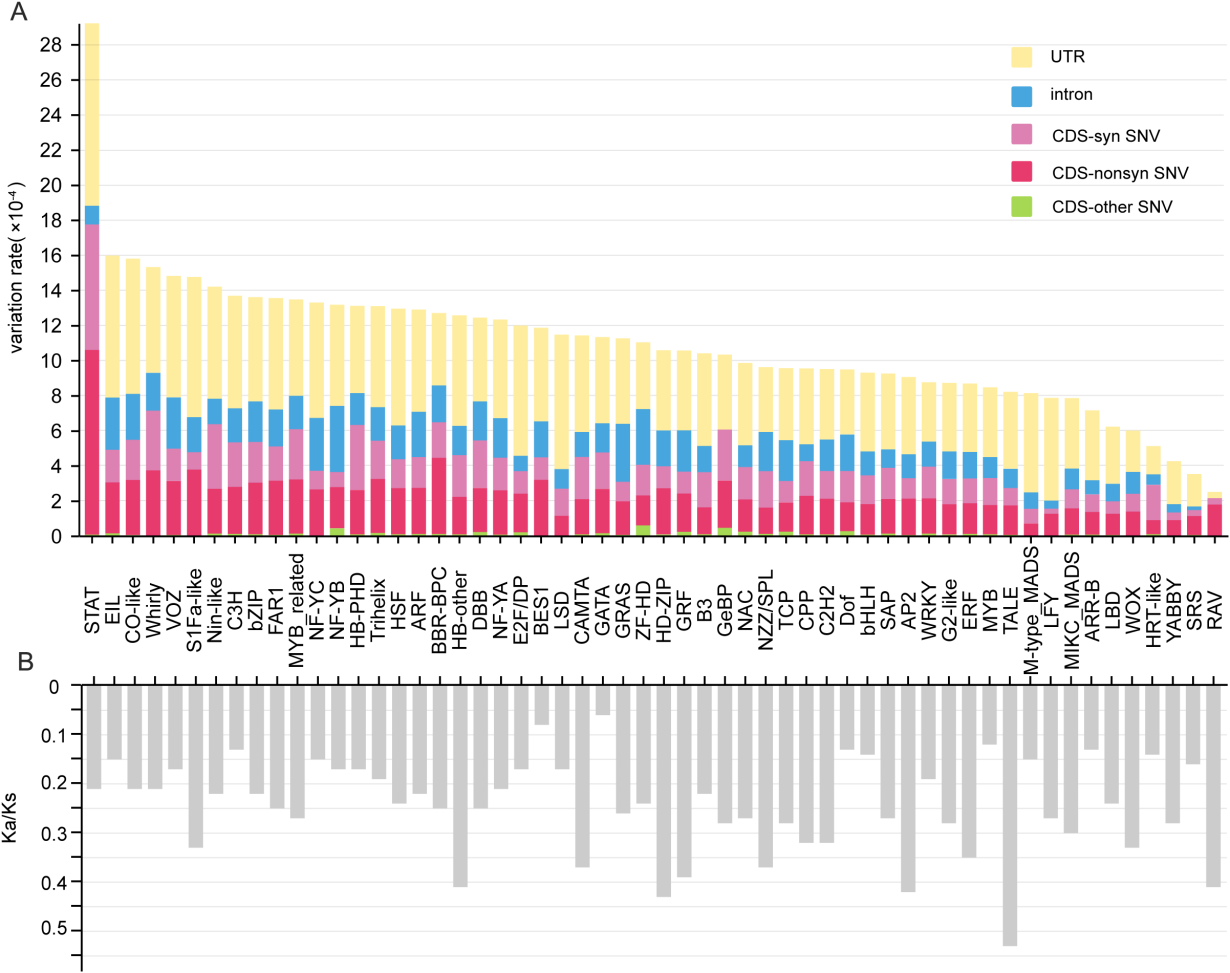
Average variation rates and Ka/Ks values of each TF family across multiple RNA-seq samples. **(A)** Average variation rates of different gene regions among each TF family. **(B)** Average Ka/Ks values of each family. CDS-non SNV indicates nonsynonymous single-nucleotide variation in the CDS region. CDS-SNV indicates nonsynonymous single-nucleotide variation in the CDS region. The other type of CDS indicates other types of variation in the CDS region.

Most (1,473, 91.9%) of the 1602 TFs had variation sites in their expressed sequences, whereas there was no variation in the expressed sequences of the remaining 129 (8.1%) TFs from 27 families (**Supplementary Table S13**). Among the 1,473 TFs with variation sites, the total number (35,508) of variation sites in the UTRs was far less than those in the CDSs (67,679) and introns (80,017) (**Supplementary Table S14**), but the variation rate of the UTRs (4.9×10^-4^) was markedly greater than those of both the CDSs (4.0×10^-4^) and introns (1.7×10^-4^) in each project sample (**Supplementary Table S15**). We further found that the variation rates in all three regions also exhibited large differences among various TF families. For example, the variation rates in CDSs, introns and UTRs varied greatly from 1.34×10^-4^ (*YABBY*) to 1.77×10^-3^ (*STAT*), from 2.1×10^-5^ (*NF-YB*) to 3.79×10^-4^ (*SRS*) and from 3.50×10^-5^ (*RAV*) to 1.04×10^-3^ (*STAT*), respectively (**Figure 5A**, **Supplementary Table S14**). Lastly, more than half of the variation sites in the CDSs were synonymous single-nucleotide variations (SNVs) (**Supplementary Table S14**). In addition, among the 27 families, the number of highly conserved members without detailed variation in *bHLH* genes reached 26, and the proportions largely varied from 1.7% in *MYB_related* to 50% in *RAV*, which consisted of only two members (**Supplementary Table S16**).

Moreover, the Ka/Ks values among the different families ranged from 0.06 (*GATA*) to 0.53 (*TALE*); most families (24, 42.9%) presented Ka/Ks values between 0.2 and 0.3, whereas a few families presented Ka/Ks values between 0 and 0.1 (*BES1* and *GATA*) and between 0.5 and 0.6 (*TALE*) (**Figure 5B**). This finding indicates that although different families have experienced strong purifying selection, selection pressure still differs significantly among different families. Notably, the Ka/Ks values of the *BES1*, *GATA*, *Dof*, *bHLH*, *MYB*, *ARR-B* and *HRT-like* families were clearly lower than those of the other families (**Figure 5B**).

### 3.8 TF expression patterns

**Figure 6:**
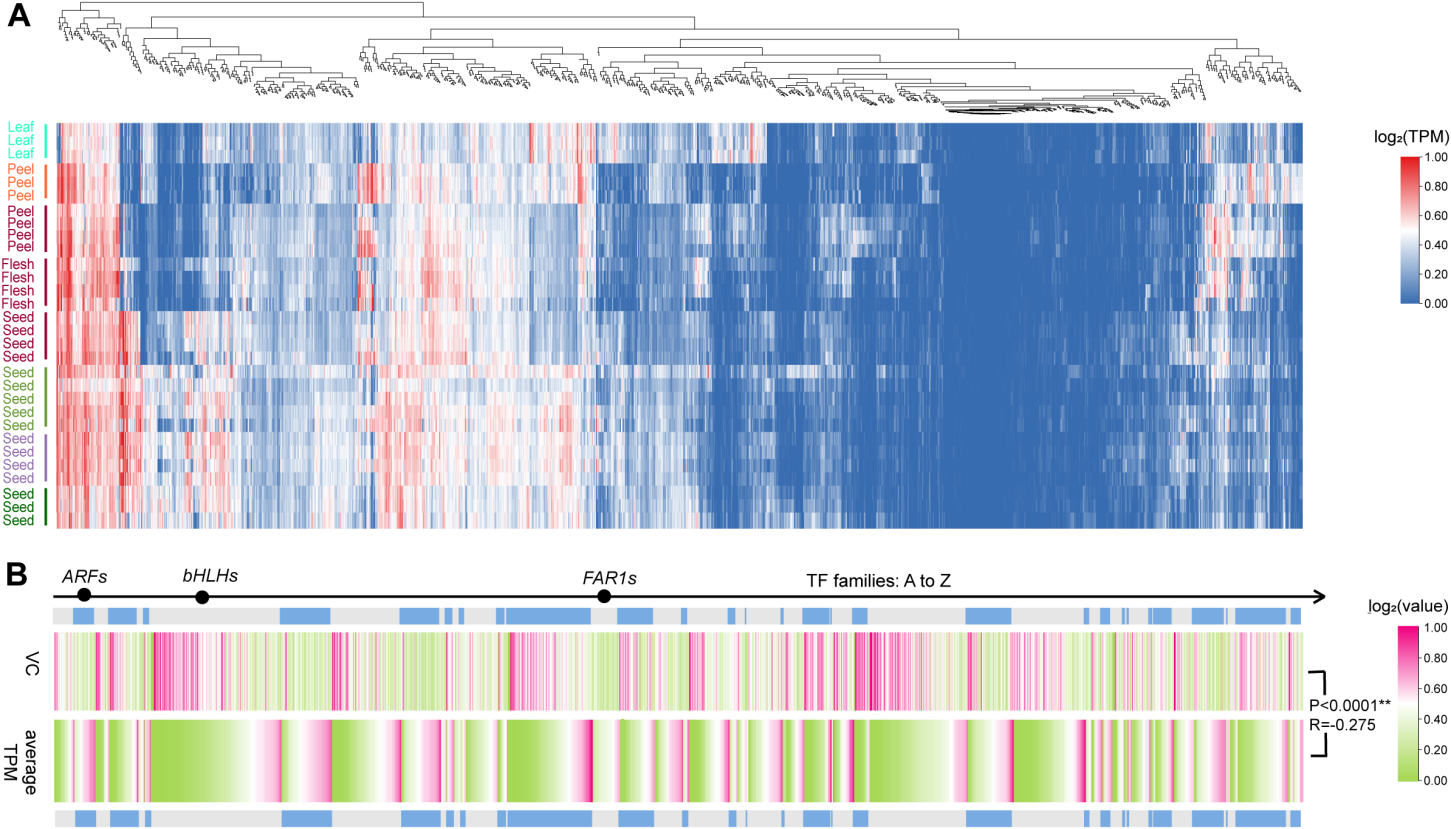
Expression patterns of TFs across integrated samples. **(A)** Clustered expression patterns in all the TFs (duplication-averaged TPM). The tissue marked with the same color is from the same NCBI project. **(B)** VCs and average TPMs across 90 samples of 1602 TFs.

The calculated expression level results revealed that 83 TFs from 28 families presented higher average TPM values of greater than 70, whereas 146 TFs from 29 families presented lower average values less than 1, and the remaining 1,373 TFs presented specific expression patterns among various samples (**Figure 6A**; **Table 17**). The average expression level of each TF varied widely among members both within and between families, and notably, there was a highly significant negative correlation (P<0.0001, R = −0.27) between the average expression levels and the CVs for all the TFs (**Figure 6B**, **Supplementary Table S17**). In addition, the VCs varied within a narrow range within some families, such as *ARF* (0.48–2.02) and *FAR1* (0.34–1.54), whereas they displayed a broader range within *bHLH* (0.36–5.48) (**Figure 6B**; **Supplementary Table S17**).

**Figure 7:**
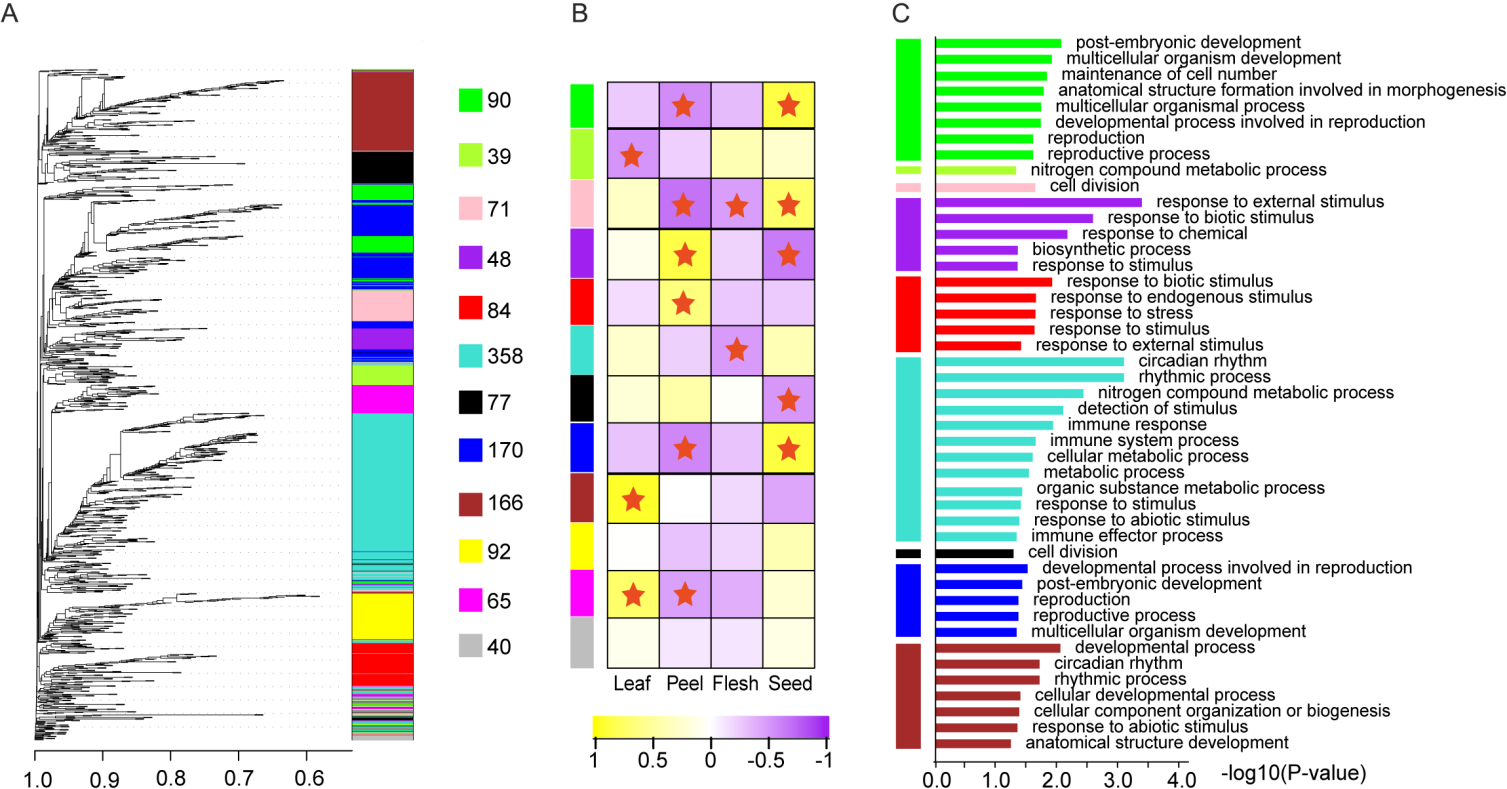
Co-expressed WGCNA module and GO function enrichment. **(A)** Co-expressing TF modules identified by WGCNA. **(B)** Tissue-specific expression pattern of each co-expressing module. **(C)** GO function enrichment of each co-expressed module.

Further weighted analysis of the gene co-expression network suggested that the 1302 TFs filtered by TPM values were clustered into 11 valid modules; the number of TFs in each module varied from 39 in the green-yellow module to 358 in the turquoise module (**Figure 7A**, **Supplementary Table S18**). Each module exhibited significant tissue-specific expression, with 1–4 modules showing high or low expression in leaves, fruit peel, and seeds, whereas only two modules presented low expression in the pulp module (**Figure 7B**). GO functional enrichment analysis revealed that all the modules were significantly enriched for functions related to “response to stimuli” and showed module-specific enrichments in functions such as “postembryonic development,” “metabolic processes,” and “rhythmic processes” (**Figure 7C**). For example, the green module, which is highly expressed in seeds, was enriched for developmental and reproductive functions, indicating its roles in individual development and organ formation. In contrast, the purple and red modules, which are highly expressed in fruit peels, were significantly enriched in stress response functions, emphasizing their roles in fruit peel stress responses. Moreover, both the purple module and the largest sky blue module were associated with metabolic processes, highlighting the essential role of transcription factors in metabolic regulation.

### 3.9 Potential cooperation of TFs

**Figure 8:**
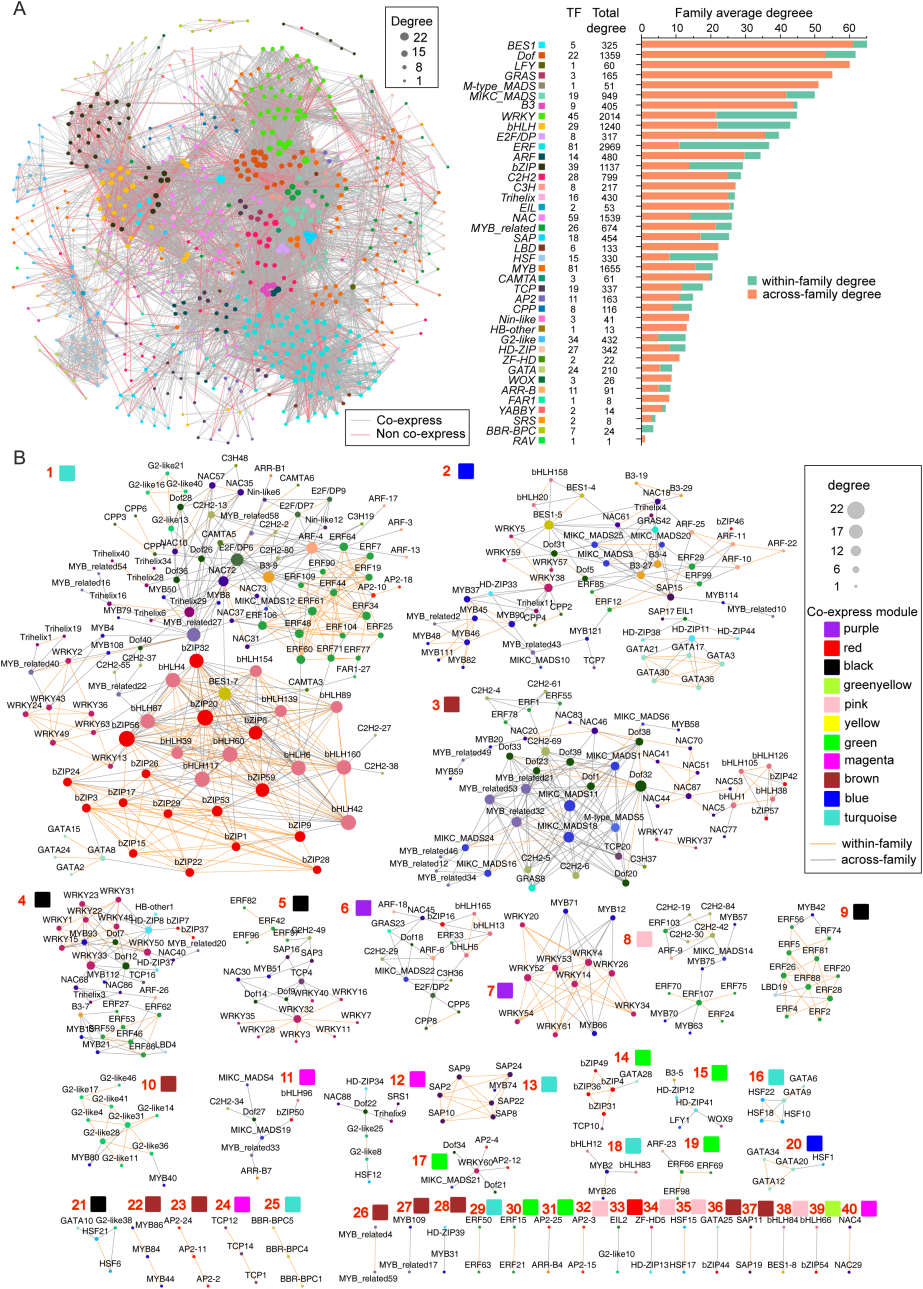
Potential cooperative TF pairs based on palindrome identification. **(A)** Potential TF pair network, in which TFs connected with red lines indicate co-expressed pairs. **(B)** Detailed co-expressing TF sets, in which the orange line indicates within-family pairs.

Based on the 704 high-confidence TFBMs (**Supplementary Table S11**), 694 TFs from 42 families could form 9,832 pairs of palindromes (from 6 bp to 21 bp) among their TFBMs (**Figure 8A**; **Supplementary Table S19**), of which 6,197 (63.1%) pairs belonged to cross-family types and 3,135 (31.9%) pairs were within-family types (**Supplementary Table S20**).

The average degree of connectivity of each family in the network significantly differed, revealing differences in the cooperation potential and cooperation mode among families. For example, the values were 65.0 in the *BES1* family but only 1.0 in the *RAV* family (**Figure 6A**). More specifically, the average degree of across-family connectivity for the *BES1, LFY, GRAS, Dof, B3*, and *MIKC_MADS* families exceeded 40.0, which was significantly greater than those of the other families (**Figure 6A**). Similarly, the *ERF, WRKY, bHLH, bZIP, HSF*, and *NAC* families presented average degrees of within-family connectivity exceeding 10.0, which were notably greater than those of the other families. Interestingly, we found a highly significant positive correlation (R=0.711, P<0.001) between family size and average within-family connectivity degree, whereas there was no significant correlation with interfamily connectivity (R=−0.119, P=0.466) (**Supplementary Table S19**). This finding indicates that larger family sizes are associated with a greater tendency for members to form dimeric interactions within the TF family.

Further comprehensive analysis of 9,833 potential dimeric cooperative relationships across RNA sequencing samples revealed 1,036 co-expression relationships involving 455 TFs from 37 families (**Figure 8B**). Of these, 605 pairs were within-family, and 431 pairs were across-family, highlighting broader cooperation across families. The TFs were organized into 40 sets, with sizes ranging from 2–94, and the degree of connectivity of individual TFs ranged from 1–22 (**Figure 8B**). Most sets contained members from multiple families, except for a few specific sets that included factors from the same family, illustrating varying cooperative potentials among transcription factors. Importantly, only 44 TFs were in simple networks with fewer than five members, whereas the majority were in more complex, highly clustered networks, demonstrating both extensive collaboration and strong connectivity among these TFs.

Additionally, the 1,036 pairs with potential dimeric co-expression relationships were distributed across 11 co-expression patterns, ranging from 1 pair in the yellow-green module to 476 pairs in the turquoise module (**Figure 7C**). This observation highlights specific biological functions that certain transcription factor combinations may regulate. For example, set 7 included 9 *WRKY* and 3 *MYB* family members, with the corresponding purple module showing specific expression in fruit peels and significant enrichment in the GO term “response to biotic stimulus”. These findings suggest that these factors may work as dimers in the disease and pest responses of *Akebia trifoliata* fruit peels. Similarly, set 14, comprising 4 *bZIPs*, 1 *TCP*, and 1 *GATA* family member, exhibited high expression in seeds and specific expression in fruit peels, along with significant enrichment in “postembryonic development”. These findings indicate that these transcription factors are likely involved in regulating postembryonic development in *A. trifoliata*.

## 4 Discussion

What enables a given organism to undertake a multitude of life activities in an orderly, effective, timely, and precise manner? The answer is that it has an omnipotent toolkit affording a sophisticated strategy to regulate gene expression at the transcriptional level, and this toolkit usually consists of hundreds to thousands of TFs. For further study of this molecular mechanism, systemically identifying all members of the toolkit in model species is crucial.

Some studies have suggested that *A. trifoliata* could play a key role in the evolutionary study of plants, especially early eudicots (Lu and Tang, 2022; Li et al., 2024), flower development, and the metabolic mechanisms of bioactive materials in fruit. In addition, biological characteristics such as a small genome size (Zhong et al., 2022a), short juvenile period for woody plants (Guan et al., 2022) and numerous seeds(Chen et al., 2023) also support the potential use of *A. trifoliata* as a model plant, especially for woody fruit crops. Recently, various scientists have focused on certain TF families, such as *Dof* (Zhang et al., 2023), *NAC* (Liu et al., 2023), *MIKC_MADS* (Zhong et al., 2022c), *WOX* (Chen et al., 2024; Han et al., 2024) and *WRKY* (Wen et al., 2022; Zhu et al., 2022), but these families are not able to explain transcription regulatory tools because they largely ignore the indispensable fact that all the TF members in a genome are functional.

In this study, a total of 1602 TFs were systemically identified in the *A. trifoliata* reference genome using a DBD scan within the PlantTFDB (**Supplementary Table S1**), which is an efficient and accurate method that has been employed to identify the TF repertoires of *A. thaliana* (Riechmann et al., 2000) and *Nicotiana tabacum* (Rushton et al., 2008) successfully. The conserved domains were classified into 56 families (**Table 1**), and the numbers of *Dof*, *NAC*, *MIKC_MADS*, *WOX* and *WRKY* members were highly consistent with those that were previously reported (**Table 1**; **Supplementary Table S1**; Zhang et al., 2023; Liu et al., 2023; Zhong et al., 2022c; Chen et al., 2024; Han et al., 2024;Wen et al., 2022; Zhu et al., 2022). In addition, most TFs could be annotated in pathways related to regulation, such as GO:0065007 (biological regulation), GO:0050789 (regulation of biological process), and GO:0140110 (transcription regulator activity) (**Figure 3**; **Supplementary Tables S9**, **S10**). Both of these findings suggested that the results for the identified TF components and their classification in this study were reliable.

We found that there were large variations in structural characteristics, including gene length, exon number, GC content, amino acid number, molecular weight, isoelectric point and even secondary structure type, among various TFs, especially those from different families (**Table 1**; **Supplementary Tables S4**, **S6**). The structural diversification could be explained by their different experiences in the evolutionary river (**Supplementary Tables S7, S8**) and the numerous allelic variations among different genotypes (**Supplementary Tables S13**, **S16**). For example, the duplication type could largely influence the number of family members, especially for those families with fewer than 10 members; specifically, the members of all five families with two members were produced by dispersed duplication, whereas those of four families with eight members were produced primarily by WGD or segmental duplication (**Figure 2**; **Supplementary Table S7**). The different evolutionary experiences could result in different distributions on chromosomes and in different compartmental locations (**Figure 1**; **Table 1**; **Supplementary Tables S1, S3**); these different locations, as well as allelic variations, could be important factors driving TF functional divergence (**Figure 6**; **Supplementary Tables S9, S10**, **S17, S18**). For example, WGD or segmental duplication could result in 126 members of MYB through family expansion (**Table 1**; **Supplementary Table S7**), in which 8 members (*MYB16, MYB29, MYB34, MYB65, MYB72, MYB101, MYB116*, and *MYB122*) without any variation sites among various genotypes could maintain their basic regulatory functions (**Supplementary Table S16**). This characteristic would further provide a chance for *MYB30* and *MYB45* to gain locomotion (GO:0040011) functions through divergence (**Supplementary Table S9**). The functional divergence of these genes was also supported by their same exon number and similar gene length, secondary structure components, extracellular location and even similar variation among various genotypes (**Supplementary Table S1**; **Supplementary Table S13**).

Among TFs, the largest puzzle is how only a few TFs can effectively regulate numerous genes and how their own expression is autoregulated. The pleiotropism of TFs could provide a reasonable explanation for the contradiction in number between TFs and SGs (Ma et al., 2022), and the molecular mechanisms could vary owing to the wide range of their very short TFBM sequences (**Figure 4B**). For example, the number of MP00370s distributed from the sole TF *CPP5* in the promoter regions of SGs reached 12,308 (**Supplementary Table S11**), and the E2F/DP family, with only 11 members, was annotated in 22 KEGG metabolic pathways (**Supplementary Table S10**). In addition, rich allelic variation sites in some families, such as E2F/DP (**Supplementary Table S13**), further increase the ability to regulate numerous genes simultaneously. Lastly, the fact that most (85.7%) TFs belong to specific expression types (**Figure 6A**; **Supplementary Table S17**) is also helpful for understanding how the same TF can regulate different genes under various environmental conditions. However, the enormous cooperation of various TFs through palindrome sequences (**Figure 8**; **Supplementary Tables S19, S20**) is the most important reason for their versatile ability to control the expression of multiple genes one time.

The final concern was the mechanism by which transcription is regulated by TFs. In this study, we noted that TFs could also widely regulate other TFs in addition to SGs (**Figure 4**; **Supplementary Table S12**). Moreover, we found that 145 members from 28 families, such as *BBR-BPC* and *MIKC_MADS*, could regulate their own transcription expression in feedback loops (**Supplementary Table S12**), which indicated that they were usually located at the top positions in TF regulation hierarchies and could control the expression of downstream genes, including other TFs and SGs, by regulatory cascades.

## 5 Conclusions

In *A. trifoliata*, we found that the transcription regulatory toolkit collectively consists of 1,602 TFs of 56 families, with variations in structural characteristics, evolutionary experiences and functional assignments among various members, especially members from different families. Furthermore, pleiotropism, rich allelic variation, specific expression and wide cooperation enable these sets of TFs to accomplish the transcriptional regulation of all the genes in the *A. trifoliata* genome effectively and precisely. Ultimately, the positive feedback regulation of some TFs, especially those from the *BBR-BPC* and *MIKC_MADS* families, could provide a reasonable explanation for their hierarchy in regulatory cascades. These results pave the way for a comprehensive understanding of the *A. trifoliata* regulatory toolkit.

## Data Availability

The datasets presented in this study can be found in online repositories. The names of the repository/repositories and accession number(s) can be found in the article/**Supplementary Material**.
